# Colocation of Lipids, Drugs, and Metal Biomarkers Using Spatially Resolved Lipidomics with Elemental Mapping

**DOI:** 10.1021/acs.analchem.2c01940

**Published:** 2022-08-18

**Authors:** Holly-May Lewis, Catia Costa, Véronique Dartois, Firat Kaya, Mark Chambers, Janella de Jesus, Vladimir Palitsin, Roger Webb, Melanie J. Bailey

**Affiliations:** † Department of Chemistry, University of Surrey, Guildford, Surrey GU2 7XH, U.K.; ‡ University of Surrey Ion Beam Centre, Guildford, Surrey GU2 7XH, U.K.; § Center for Discovery and Innovation, Hackensack Meridian School of Medicine, 123 Metro Boulevard, Nutley, New Jersey 07110, United States; ∥ Faculty of Health and Medical Sciences, University of Surrey, Guildford, Surrey GU2 7XH, U.K.

## Abstract

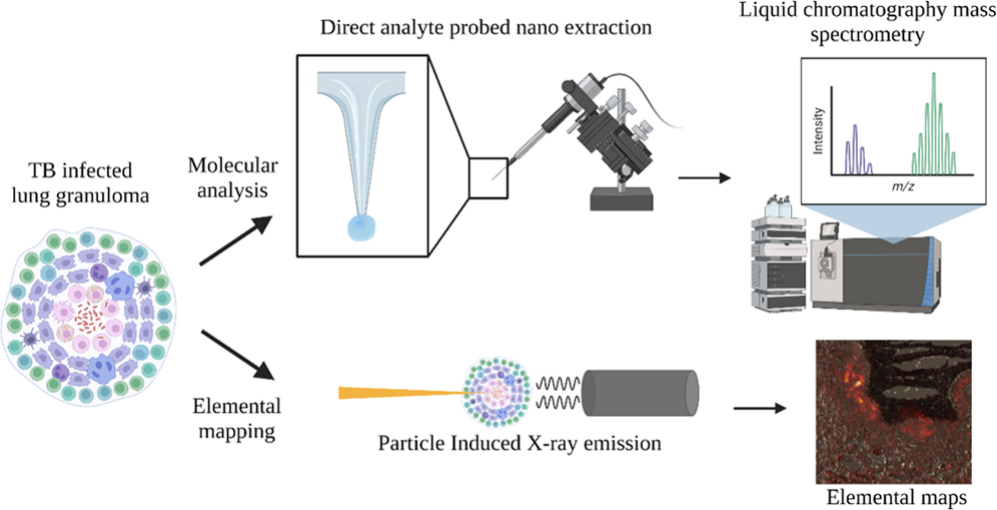

Elemental imaging is widely used for imaging cells and tissues but rarely in combination with organic mass spectrometry, which can be used to profile lipids and measure drug concentrations. Here, we demonstrate how elemental imaging and a new method for spatially resolved lipidomics (DAPNe-LC-MS, based on capillary microsampling and liquid chromatography mass spectrometry) can be used in combination to probe the relationship between metals, drugs, and lipids in discrete areas of tissues. This new method for spatial lipidomics, reported here for the first time, has been applied to rabbit lung tissues containing a lesion (caseous granuloma) caused by tuberculosis infection. We demonstrate how elemental imaging with spatially resolved lipidomics can be used to probe the association between ion accumulation and lipid profiles and verify local drug distribution.

## Introduction

Elemental mapping has demonstrated considerable utility in biomedical research,^1^ for example, to probe metalloproteins,^2^ environmental pollutants,^3^ or drug uptake,^4^ and to understand the pathogenesis of diseases such as Alzheimer’s,^5^ cancer,^6^ and diabetes^7^ in tissues. A number of elemental imaging modalities exist, mostly based on X-ray or mass spectrometry. These have complementary characteristics, including elemental coverage, quantitation, detection limits, and spatial resolution, and include X-ray fluorescence (XRF),^8^ scanning electron microscopy energy dispersive X-ray analysis (SEM-EDS),^9^ particle-induced X-ray emission (PIXE),^10,11^ secondary ion mass spectrometry (SIMS),^12^ laser ablation inductively coupled plasma mass spectrometry (LA-ICP-MS),^10^ and laser-induced breakdown spectroscopy (LIBS).^13^

Due to the importance of elemental mapping in biomedical research, there is increasing interest in their use in combination with molecular imaging.^14^ Molecular imaging approaches such as matrix-assisted laser desorption ionization (MALDI), desorption electrospray ionization (DESI), and secondary ion mass spectrometry (SIMS) are steadily gaining momentum in biomedical science.^15^ These tools can be used to probe localized drug, metabolite, and lipid distributions to gain a better understanding of drug–host interactions,^16,17^ drug–pathogen interactions,^18^ and disruption to biological pathways.^19^ Elemental and molecular correlative imaging is steadily gaining momentum and could be used to colocate elemental and molecular biomarkers, or to unravel the regulatory mechanisms involved in trace metal transport, storage, and distribution.^1^ Combining approaches in this way can be of particular interest to probe the local chemical environment when the system under exploration includes a metal-containing drug,^20^ protein,^21^ pollutant, or metal accumulation by the host.^22^

A small number of recent publications have reported multimodal imaging to correlate elemental and molecular markers. These include imaging sequential sections of tissues using LA-ICP-MS with DESI,^23^ MALDI,^24^ and SIMS.^25^ Attempts have also been made to coregister elemental and molecular markers on the same tissue section, to enable more accurate relocation of features, via LA-ICP-MS-MALDI,^20,21^ XRF-MALDI,^26^ and PIXE-DESI.^22^ The dearth of literature in this domain demonstrates that correlative imaging of molecular and elemental markers is still relatively immature. This is because the optimal combinations of techniques are not established yet, and in addition to this, the sample-handling requirements can be different, the techniques operate at different length scales, the facilities and expertise for these two approaches are not always colocated, and no framework for data integration exists.

One of the disadvantages of mass spectrometry imaging is that the sample removal and ionization processes are coupled together. This carries several disadvantages, including ion suppression, which can cause matrix effects, and limits the coverage (sensitivity) to biomolecules.^27^ An alternative approach to mass spectrometry imaging is liquid extraction surface analysis, where a discrete region of a sample is probed.^28−30^ Here, sample removal and ionization are decoupled, giving the opportunity to remedy some of the disadvantages of mass spectrometry imaging. In the liquid extraction approach known as direct analyte-probed nanoextraction (DAPNe), a nanospray capillary filled with a solvent is directed at an area of interest on a sample, the solvent is pushed onto the surface to dissolve analytes, and these are aspirated into the capillary tip. Unlike mass spectrometry imaging, DAPNe provides the opportunity for chromatographic separation, to separate isobaric compounds and reduce ion suppression effects.^31^

In this work, we have developed spatially resolved lipidomics using DAPNe. We show how spatially resolved lipidomics, followed by elemental mapping, can be used to correlate metal accumulation with localized lipid profiles in rabbit lung tissues, following tuberculosis infection. The elemental mapping is carried out post-DAPNe, and therefore, we consider this workflow to be suitable for a wide range of elemental imaging modalities. In this work, the ion beam analysis technique PIXE is used to provide elemental maps.

Ion beam analysis (IBA) is a suite of elemental mapping techniques, which uses an MeV ion beam to probe the sample. PIXE is an IBA technique that offers parts per million sensitivity and submicron spatial resolution.^11^ A unique aspect of ion beam analysis is that the backscattered particle spectra generated by IBA techniques can simultaneously provide information on elemental depth distributions, light elements (C, N, O), and sample thickness. This information can be used to account for X-ray absorption and therefore correct for matrix effects in the X-ray images.^10^ Unlike LA-ICP-MS, XRF, SEM-EDS, and SIMS, matrix-matched samples are not required for quantification.^32^ We show how DAPNe and PIXE, when used in combination, can act as orthogonal methods to verify localized drug distribution.

## Materials and Methods

### Sample Preparation

#### Homogenized Tissue

Liver tissue was obtained as surplus control material from untreated, nongenetically modified mice used for other studies, approved by our local ethical committees, from the Biomedical Research Facility of the University of Surrey. In accordance with 3Rs principles, no animals were sacrificed solely for the purpose of the work described here. Liver homogenates were used for DAPNe-LC-MS method optimization and were prepared as described by Swales et al.^33^ Liver tissue was homogenized and pipetted into molds (2 mL Pasteur pipette bulb) and then frozen at −80 °C. Samples were sectioned to 10 μm thickness using a Thermo NX70 Cryostar (ThermoScientific, Germany) and thaw-mounted onto glass slides before being vacuum packed and stored at −80 °C. Samples were brought to room temperature in the vacuum packing prior to analysis.

#### Fresh Frozen Lung Tissue

Lung tissue was collected from *Mycobacterium tuberculosis*-infected rabbits, handled, and processed in the BSL3 in compliance with protocols approved by the Institutional Biosafety Committee of the National Institute of Allergy and Infection Disease, NIH, and Hackensack Meridian Health, NJ, from studies approved by the Institutional Animal Care and Use Committee of the National Institute of Allergy and Infection Disease, NIH, Bethesda, MD (protocol number LCIM-3). All studies followed the guidelines and basic principles stated in the United States Public Health Service Policy on Humane Care and Use of Laboratory Animals.

Female New Zealand White (NZW) rabbits weighing 2.2–2.6 kg were maintained under specific pathogen-free conditions and fed water and chow *ad libitum*. NZW rabbit ID 713 was infected with *M. tuberculosis* HN878 using a nose-only aerosol exposure system as described.^34^ At 12 weeks post infection, once mature cellular and necrotic lung lesions had developed,^35^ the rabbit received 28 daily doses of bedaquiline at 20 mg/kg. Twenty-four hours after the last dose, lung lesions embedded in the surrounding tissue were collected for imaging and snap-frozen in liquid nitrogen vapor as described previously.^36^ To sterilize the samples and inactivate all viable *M. tuberculosis* bacilli, samples were irradiated in a Co-60 irradiator until the exposure reached 3 Mrad (validated as a sufficient exposure to kill all viable *M. tuberculosis* bacteria present in lung lesions). Dry ice was resupplied as required to keep the samples frozen at all times. The frozen rabbit lesions were sectioned at 10 μm thickness using a CM1860 UV cryostat (Leica) at −20 °C. The sections were thaw-mounted onto 1.4 μm thick PET membrane slides (Leica), shipped on dry ice, and stored at −80 °C.

### Materials

The solvents used to prepare the solutions and solvent mixture (methanol (MeOH), ethanol (EtOH), acetonitrile (ACN), water (H_2_O), isopropanol (IPA), and formic acid (FA)) were Optima LC-MS-grade obtained from Fisher Scientific, Loughborough, U.K. Bedaquiline (>97% purity) was obtained from Sigma-Aldrich, Poole, U.K. For administration to rabbits, a high-purity fumarate bedaquiline salt was obtained through the NIH/ATCC, through the HIV Reagent Program (https://www.hivreagentprogram.org). Certified reference material (>97% purity) of the antibiotic drug analyte, bedaquiline, was obtained from Sigma-Aldrich.

### Direct Analyte-Probed Nanoextraction (DAPNe)

An upright (Nikon AZ100) microscope was used to view the tissues using transmitted light. Gold-coated glass capillary CT2 tips, with an internal diameter of 3–5 μm (Yokogawa, Japan), containing 5 μL of the extraction solvent (50:50 MeOH/EtOH) were guided to the area of interest using a nanomanipulator (Attocube, Germany). The injection and reaspiration of the solvent were controlled by a PM2000 microinjector (MicroData Instrument, Plainfield, NJ). The extraction solvent was injected at a pressure of 1 psi for 0.1 s. A 0.8 psi balance pressure was applied and the solvent was dwelled on the surface for ∼30 s and was aspirated back into the tip for 1 s. The injection of the extraction solvent leaves a visible extracted region on the sample. The area of the extraction area was measured using the NIS-D Elements software (Nikon, Japan).

### LC-MS

Samples were introduced to LC immediately following extraction using DAPNe, by positioning the capillary tip over an LC-MS vial and injecting the contents of the tip into the vial using a gas syringe combined with a tip holder. Samples were shaken using a vortex mixer for 30 s to ensure a uniform composition. The sample (5 μL) was then injected into the LC. Liquid chromatography analysis was conducted on an Ultimate 3000 UHPLC system (ThermoScientific, Bremen, Germany). The analytes were separated using a Kinetex C18 column (100 × 2.1 mm^2^, 1.7 μm) at a flow rate of 0.3 mL/min and a column temperature of 55 °C. The mobile phases were solvent A 60:40 acetonitrile/water and solvent B 90:10 isopropanol/acetonitrile. Both the mobile phases had 0.1% formic acid added to aid ionization. The initial mobile phase was 60% solvent A and 40% solvent B, which was modified to 50% solvent A and 50% solvent B over 1 min. Solvent B was then increased to 69% over 2.6 min and increased further to 88% over the next 8.4 min. The mobile phase composition was then returned to the initial mobile phase over 2 min and was kept constant for 2 min. The overall run time was 16 min. The UHPLC system was coupled to a Thermo Orbitrap Q-Exactive Plus mass spectrometer. The electrospray ionization source was optimized and operated with a spray voltage of 3 kV and a capillary temperature of 300 °C. Data were acquired at a mass range of 100–1200 *m*/*z* with a mass resolution of 70 000 (at *m*/*z* 200) with the automatic gain control (AGC) on and set to 1e6 ions.

### Data Processing

To analyze the lipid data, ThermoScientific LipidSearch software was used to process the LC-MS data and identify lipid peaks. To compare different regions of samples, SIMCA (MKS Umetrics) was used to carry out partial least-squared discriminant analysis (PLS-DA). Prior to analysis, the data were normalized to the spot area, log transformed, and pareto scaled. The PLS-DA analysis gave a list of lipids and their corresponding variable importance in projection (VIP). A VIP score is a measure of a variable’s importance in the PLS-DA model. It gives the contribution each lipid makes to the model; therefore, the higher the VIP score, the more it contributes to the model.

### PIXE

Elemental mapping was carried out using particle-induced X-ray emission (PIXE) at the University of Surrey Ion Beam Centre, U.K. Samples were analyzed using 2.5 MeV protons, with beam currents ranging from 300 to 600 pA. The beam was focused to ∼2 × 2 μm^2^ (measured using a 1000 copper grid). The scan size was 2 × 2 mm^2^ for the PIXE maps presented in Figure 3. PIXE maps presented in Figure 6 were obtained using a mosaic mode, where six 1 × 1 mm squares were analyzed in a 2 × 3 grid arrangement.

X-rays were detected using a silicon drift detector (RaySpec, U.K.) with an active area of 80 mm^2^, mounted at a central angle of 135° to the beam direction in the horizontal plane, and with a 130 μm beryllium (Be) foil to stop backscattered particles reaching the detector. Backscattered particles were detected using a PIPS charged particle detector (Mirion Technologies) with an active area of 150 mm^2^. The X-ray and backscattered particle detector responses were calibrated using a BCR-126A glass standard (European Commission, Joint Research Centre (JRC), Geel, Belgium). Data were acquired and analyzed using OMDAQ-3 software (Oxford Microbeams, Ltd., U.K.).

## Results

### Spatially Resolved Lipidomics

**Figure 1:**
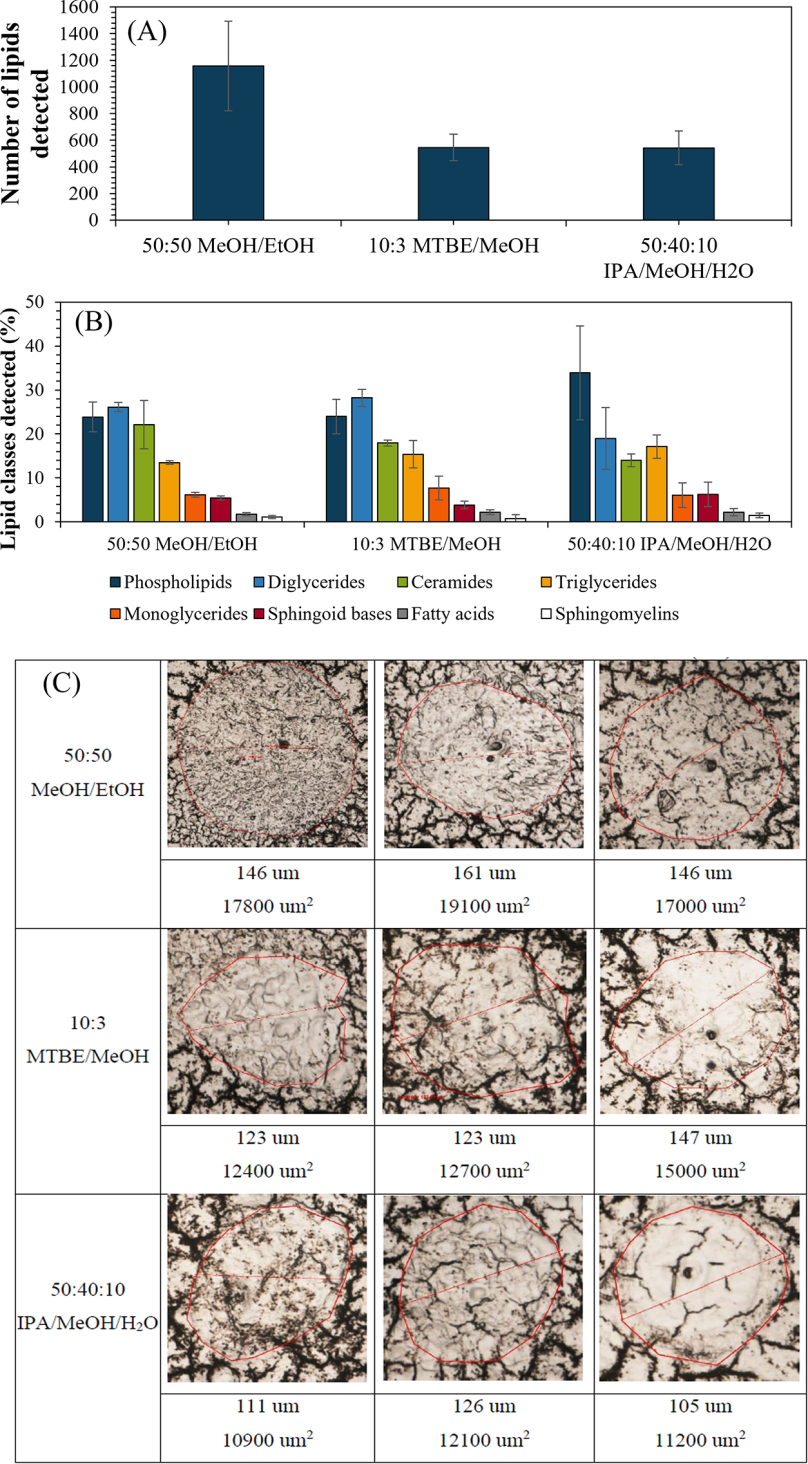
Comparison of different extraction solvent systems for DAPNe-LC-MS (*n* = 3 per solvent) using a homogenized liver sample. (A) Number of lipid features detected, (B) proportion of lipids detected by class, and (C) optical images of tissue sections post extraction using different solvent systems. Error bars represent 1 standard deviation. Red circles are used to highlight the wetted area of the tissue assumed to represent the sampling area.

Figure 1 summarizes the performance of a DAPNe-LC-MS method for spatially resolved lipidomics. This is the first time DAPNe-LC-MS has been used for lipidomics analysis, and it was therefore necessary to explore the optimal sampling conditions. To do this, we chose to optimize the method using homogenized mouse liver, which has been reported in previous works^28,37^ and gives a spatially uniform sample rich in lipids. Three extraction solvents were tested for suitability, based on a literature search of untargeted lipidomics methods. For each test extraction solvent, three repeat extractions from tissue homogenates were carried out using the DAPNe-LC-MS method described above. The number of lipid features identified by the LipidSearch software is presented in Figure 1A, the number of lipids assigned and detected in each class are presented in Figure 1B and the microscope images showing the extraction areas are shown in Figure 1C. While the IPA/MeOH/H_2_O solvent gave the smallest spot areas of the solvent systems tested, the MeOH/EtOH extraction solvent gave the highest number of lipid features (ions detected and classified as lipids by the LipidSearch software) per extraction (*P* < 0.05), indicating that the lipid coverage was best with this solvent. Lipid coverage was prioritized over spatial resolution and therefore MeOH/EtOH was adopted for further measurements. This was done to maximize the complementarity between this approach and mass spectrometry imaging, which has good spatial resolution but suffers from gaps in coverage.

**Figure 2:**
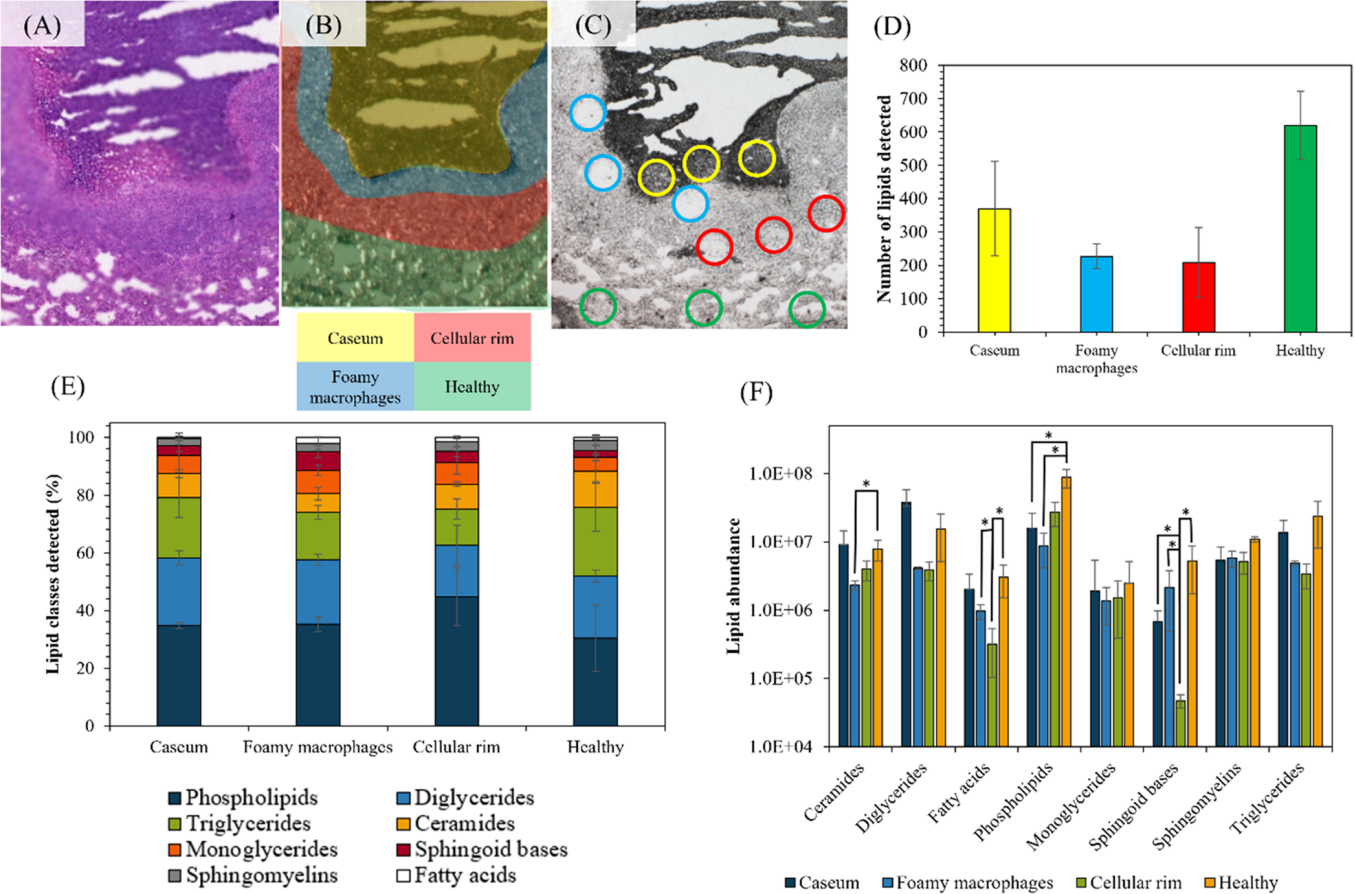
(A) Annotated H&E image of a rabbit lung tissue section containing a lesion produced by *M. tuberculosis*; (B) annotated microscope image of a TB granuloma color-coded according to pathological features: central caseum is the result of necrosis of infected foamy macrophages that form the innermost layer of the cellular region, the area annotated as “cellular rim” contains a majority of lymphocytes; (C) DAPNe extraction spots color-coded by the tissue region; (D) bar chart showing the number of lipids detected in each of the annotated regions; (E) relative abundance of lipid profiles for each tissue region; and (F) graph showing the total peak intensity per lipid class; the brackets show where there is a significant difference (*P* < 0.05) (full table shown in Figure S2).

In Figure 2A, a microscopic H&E image of a rabbit lung tissue section containing a lesion produced by *M. tuberculosis* is presented. Previous work has shown that these types of lesions (caseous granuloma) contain spatially organized immune cell types surrounding a necrotic center also called “caseum”,^38^ and the key tissue regions of interest (ROIs) are annotated (Figure 2,B). The rabbit analyzed here presented extensive immunopathology with several large necrotic lesions and several cavities. We chose a large caseous lesion to illustrate our analytical approach because it contains the major and distinct sites of infection, namely: (a) the lymphocyte- and neutrophil-rich cellular rim, where bacteria are mostly intracellular; (b) the thin foamy macrophage layer, where again bacteria are intracellular and possibly more persistent; and (c) the necrotic/caseous center “caseum”, where bacteria are extracellular and extremely drug tolerant.

Three repeat samples were taken from each ROI using DAPNe, as illustrated in Figure 2C (see Figure S1 for post extraction optical images). DAPNe-LC-MS was able to detect 209–620 lipid features in different ROIs, as shown in Figure 2D. Significantly fewer lipid species were detected in the ROIs corresponding to the diseased tissue compared to the healthy one (confirmed by Mann–Whitney *U* test, *P* < 0.05). Figure 2E,F shows the lipid profiles detected in each region using the classification assigned by LipidSearch, expressed as relative and absolute abundances, respectively. Most notably, the cellular rim had the lowest abundance of sphingoid bases, significantly less than all of the other ROIs.

**Figure 3:**
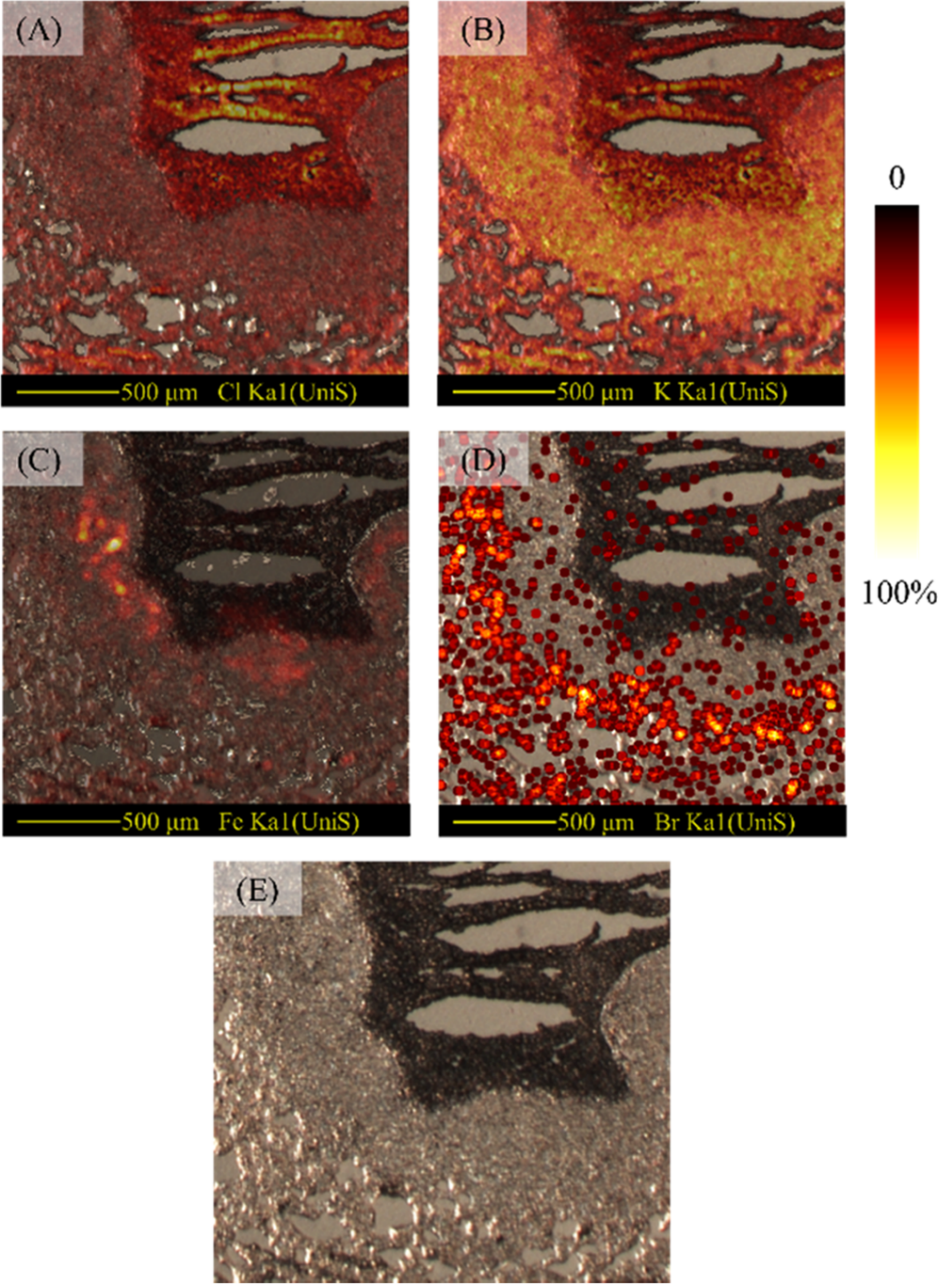
PIXE maps of (A) chlorine, (B) potassium, (C) iron, and (D) bromine, showing a 2 × 2 mm^2^ region in a selected region of rabbit lung, containing a lesion identified as a caseous granuloma, and (E) a reference microscope image of the tissue.

A sequential section of the tissue was imaged using PIXE and Figure 3A–D shows the distribution of the elements Cl, K, Fe, and Br, respectively. The periphery of the caseum region was used to align the ion beam and microscope images and relocate the DAPNe extraction areas, as shown in Figure 2C. Comparing the PIXE Fe map of Figure 3C with the annotated regions shown in Figure 2A,B reveals that the foamy macrophage ROI is associated with the accumulation of Fe. According to the spatially resolved DAPNe-LC-MS data (Figures 2F and S2), this region has the lowest abundance of ceramides of all regions, significantly lower than the healthy region (confirmed with Mann–Whitney *U* test *P* < 0.05).

**Figure 4:**
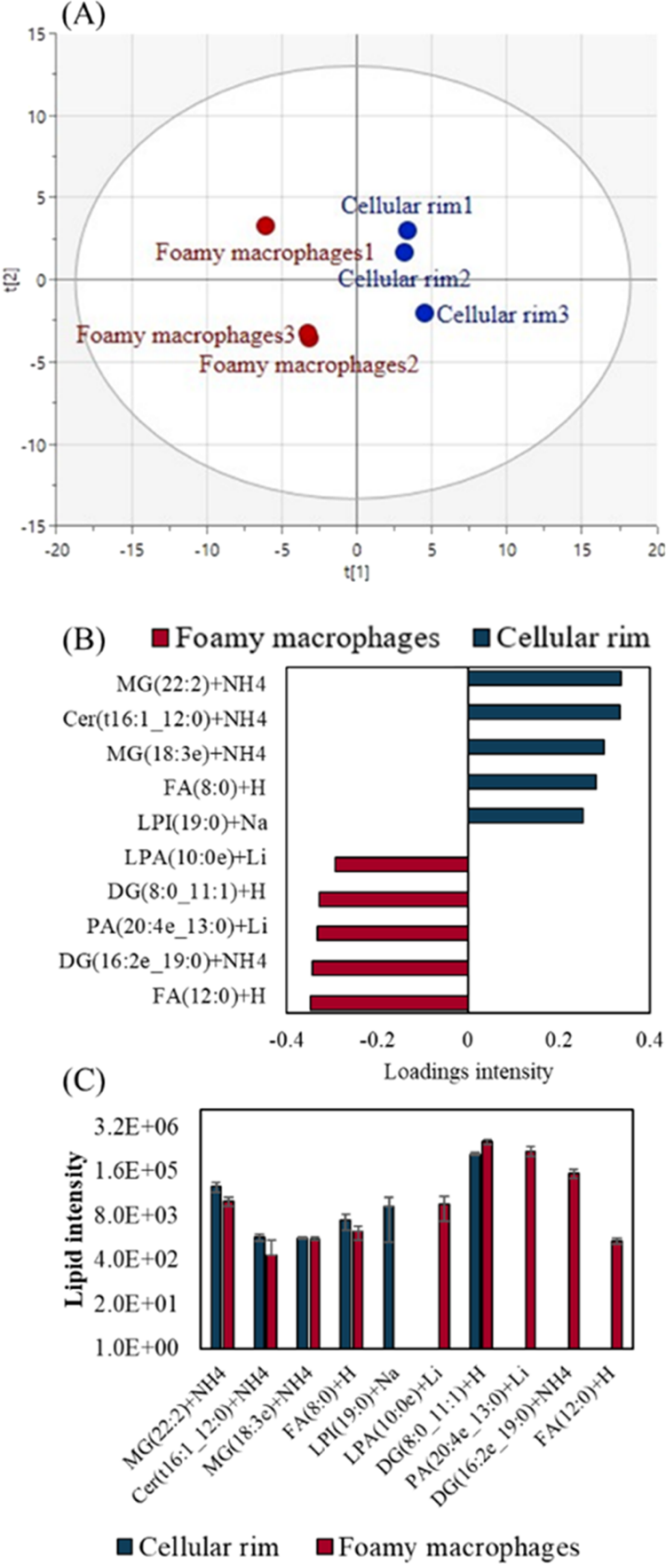
(A) PLS-DA of the foamy macrophage vs the cellular rim with scores for the three regions of each sample (R2Y = 0.99, Q2Y = 0.68), (B) top VIP scores for the two ROIs, and (C) bar chart of VIP scores in the two ROIs.

To further explore the association between Fe accumulation and lipid profiles, we used partial least-squares discriminant analysis (PLS-DA) to screen for differences in lipid signals between the foamy macrophage (elevated Fe) and the neighboring cellular rim region. In Figure 4A, the PLS-DA model used to capture differences between the cellular rim and the foamy macrophage region is presented, and Figure 4B shows the top 10 VIP scores. In Figure 4C, the intensity of the top VIP scored features are plotted for each of the two ROIs. Peaks assigned to FA, PA, LPA, and DG were found to be associated with the (high Fe) foamy macrophage region. One lipid that was associated only with the cellular rim was LPI(19:0) (lysophosphatidylinositol). Interestingly, phosphatidylinositols, including LPI(19:0)-containing tuberculostearic acid, have recently been shown to be reliable lipid markers of mycobacterial load.^39^

**Figure 5:**
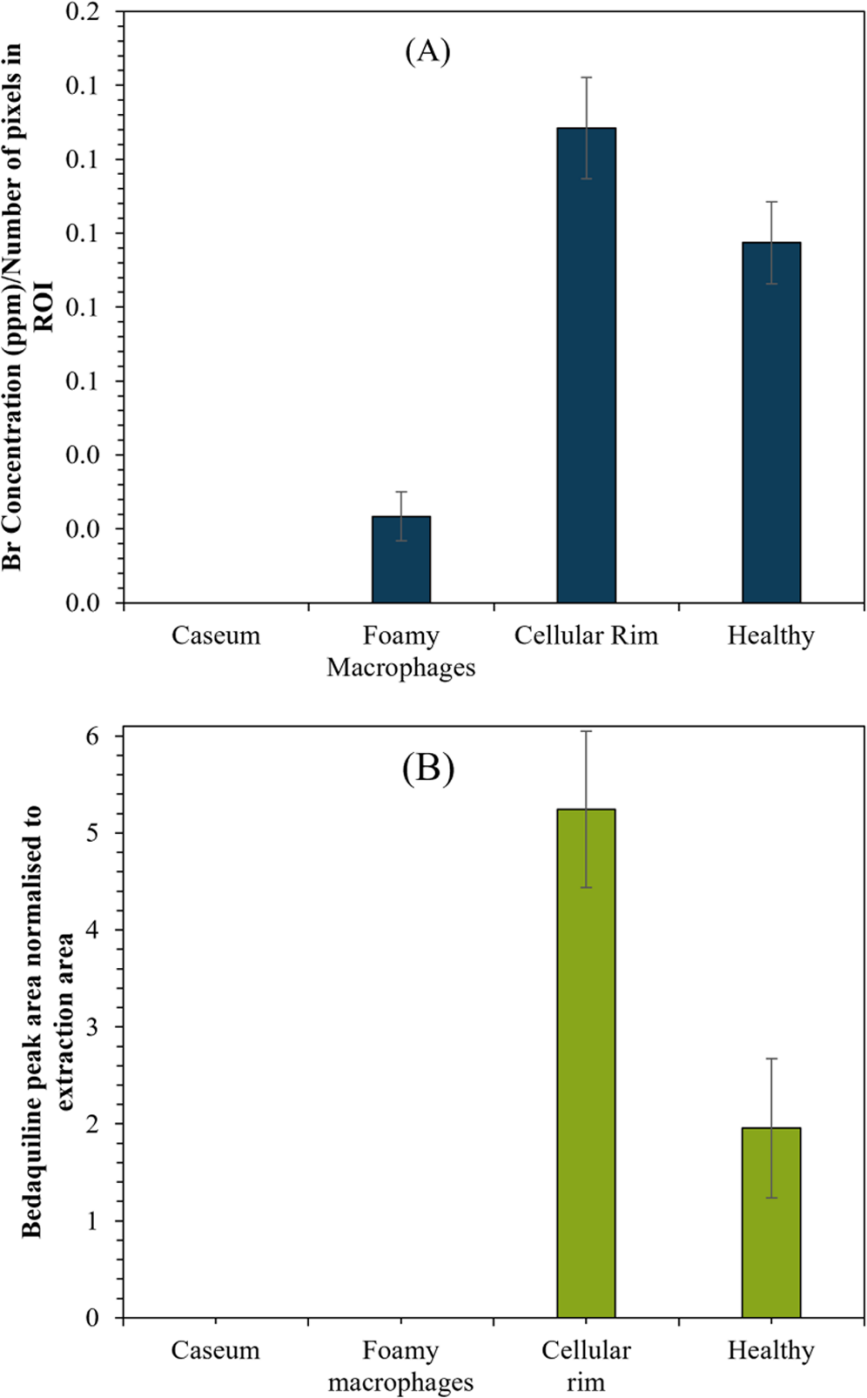
Chart showing (A) Br peak area as measured by PIXE normalized to the area in granuloma ROIs and (B) Bedaquiline peak area (normalized to the extraction area) as measured by DAPNe-LC-MS.

The rabbit used in this study had been dosed with the Br-containing anti-TB drug bedaquiline (the structure is shown in Figure S3). It has been demonstrated previously that bedaquiline can be identified through imaging of Br using SIMS.^40^ In Figure 3D, the Br map produced by PIXE imaging is presented. The PIXE image suggests that bedaquiline is most concentrated to the cellular rim. This was supported by the calculation of Br concentration (normalized to the number of pixels in the regions of interest) as measured by PIXE (Figure 5A). Tissue from a second animal, used as a bedaquiline-free control, was imaged using PIXE, and Br was not detected, showing selectivity to bedaquiline (data not shown).

We have confirmed these findings using DAPNe-LC-MS by detecting the protonated molecular ion of bedaquiline (*m*/*z* 555.1642). Peak assignment was confirmed by the retention time using a bedaquiline certified reference material. Figure 5B shows the normalized (to spot area) intensity of bedaquiline detected by DAPNe-LC-MS in each granuloma ROI, showing that the drug concentration is indeed highest in the cellular rim and then in the healthy tissue but below the limit of detection by DAPNe-LC-MS in the regions of caseum and foamy macrophages. This demonstrates for the first time that DAPNe-LC-MS has sufficient sensitivity to probe therapeutic drug concentrations in tissues, although the PIXE data indicate even greater sensitivity of detection (Figure 5A).

### Sequential Measurements

**Figure 6:**
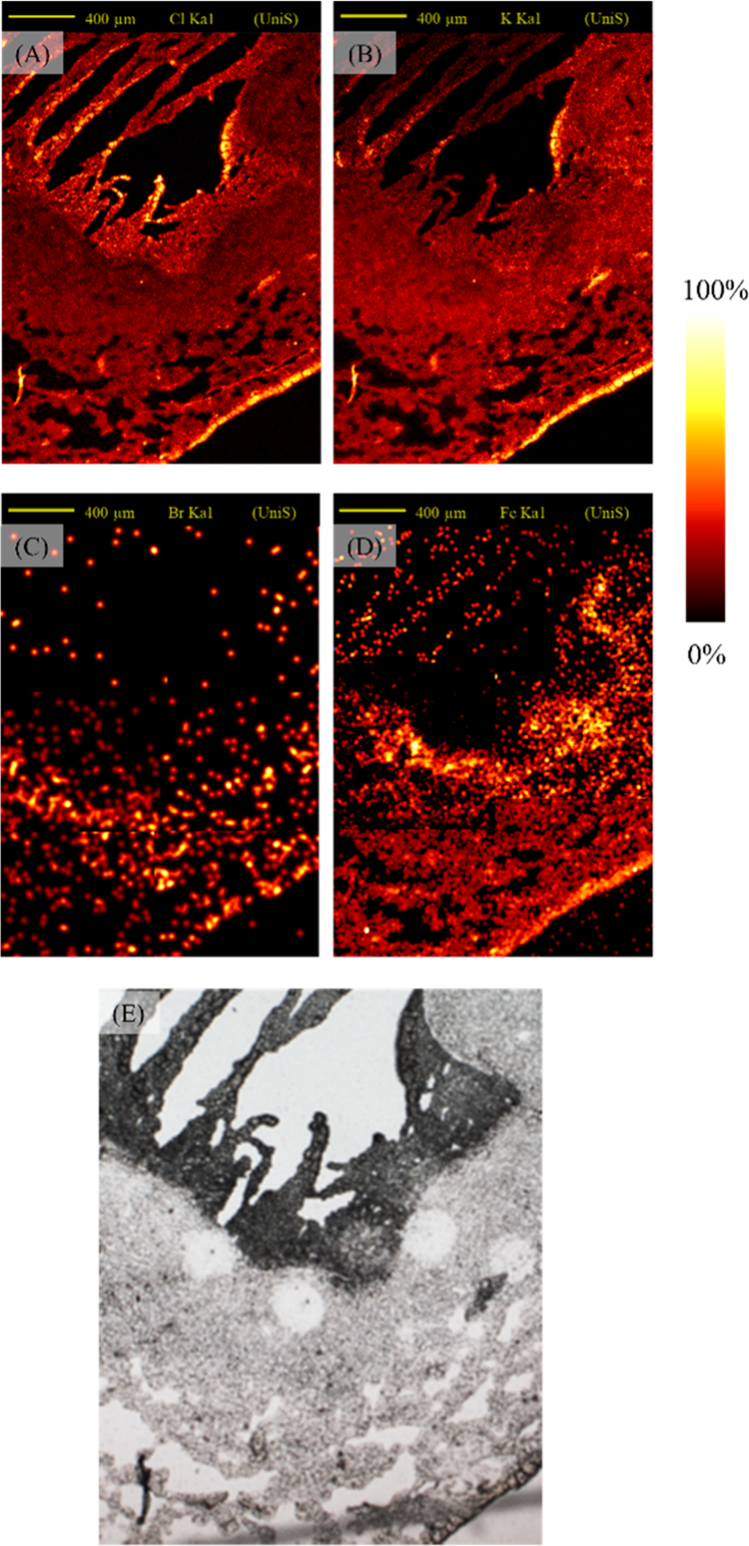
PIXE maps for (A) chlorine, (B) potassium, (C) bromine, (D) iron, and (E) optical image of TB granuloma following DAPNe extractions.

In a previous work, we have demonstrated sequential imaging of elemental and chemical species on the same tissue section using PIXE and DESI.^41^ However, one limitation of this approach was that mobile ions were delocalized by the DESI solvent probe. To explore the possibility of correlating lipids and elements on the same tissue section, the tissue from Figure 2 is analyzed post-DAPNe measurement, and is shown in Figure 6A–D. Although the areas measured by DAPNe are observable from the microscope images as paler spots (Figure 6E), DAPNe has not affected the trace element images. This is evidenced by very similar images to those generated pre-DAPNe (through comparison of Figures 3A–D and 6A–D).

## Discussion

The objective of this study was to demonstrate that elemental imaging (PIXE) and a new method for spatially resolved lipidomics (DAPNe-LC-MS) can be used in combination to probe the relationship between metals, drugs, and lipids in discrete areas of tissues. This work has shown that it is possible to both visualize and quantify differences in lipid profiles and trace elements in different regions of a tuberculosis lung granuloma. We found that Fe accumulates in the foamy macrophage region of the granuloma tissue, associated with higher amounts of FA, PA, LPA, and DG than the adjacent cellular rim, where Fe did not accumulate. These observations are consistent with (1) the accumulation of triacylglycerol-rich lipid bodies as characteristic of foamy macrophages;^42^ (2) the synthesis of triacylglycerol, following the pathway LPA → PA → DG; and (3) the promotion of both fatty acid import into cells and lipid droplet formation by Fe (reviewed in Rockfield et al.^43^).

More surprising was the finding that the foamy macrophage regions we sampled had the lowest abundance of ceramides of all regions, significantly lower than the healthy regions. An increase in intracellular iron content normally follows ceramide accumulation (promoting hepcidin expression) with a consequent increase in further ceramide production (via activation of sphingomyelin hydrolysis^43,44^), but the opposite association appears to be the case in the tissue we sampled.

Several other lipids were found to be elevated in the foamy macrophage region compared with the neighboring cellular rim—including sphingoid bases and fatty acids. Previous work employing bulk measurements of tissues and cells has pointed to an association between iron accumulation and sphingolipids in (other) biological systems.^44^ There is also evidence of an association between Fe and fatty acid import and metabolism.^43,45^ A combination of elemental imaging with lipidomics analysis therefore allows these pathways and biological processes to be explored in spatial compartments in tissues for the first time. The power to detect genuine associations between the spatial accumulation of particular elements and the associated abundance of specific lipids would be expected to increase as the sample size increases (i.e., number of animals, tissue sections, area of each tissue/region).

In PIXE imaging, matrix artefacts occur (and can be corrected for) when the elemental composition of a material changes substantially.^11^ This is because the elastic backscattering (EBS) spectra, collected simultaneously with the PIXE spectra, changes according to the major element composition and thickness of the tissue. The EBS images therefore provide a monitoring tool for matrix artefacts and thickness variation. As expected for a thin, soft tissue sample, the EBS images were uniform across the tissue (see Figure S4), showing that the major element (C, N, O) composition was uniform. The Br PIXE images therefore show the true distribution of bedaquiline. In contrast, the DAPNe lipidomics analysis (Figure 2) demonstrates how the chemical composition of the tissue changes in different granuloma ROIs. It is well established that mass spectrometry imaging methods are prone to matrix artefacts arising from ion suppression or enhancement.^46^ This can make it difficult to establish the true distribution of a drug in a tissue. We therefore propose that PIXE imaging can provide a sensitive and alternative means of imaging the distribution of metal-containing drugs in tissues where the matrix changes substantially, and that this measurement can be complemented by mass spectrometry to confirm peak assignment.

The DAPNe-LC-MS approach was designed to reduce ion suppression effects by providing separation (through chromatography) prior to mass spectrometry analysis. Indeed, the LC-MS method gave good precision and a linear response to standards (see Figure S5). On inspecting Figure 5, there is a broad agreement between PIXE and DAPNe on the drug distribution, with bedaquiline concentrating in the cellular rim, consistent with previous observations.^47^

It was found that PIXE and DAPNe-LC-MS can be used in sequence. The chosen solvent (50:50 MeOH/H_2_O) was observed not to delocalize any elemental species and therefore PIXE analysis could be carried out after DAPNe analysis. Figure S6 shows that this is solvent-dependent—if a water:methanol solvent is used for DAPNe, the delocalization of elemental species is clearly visible.

## Conclusions

We have developed a new method for spatially resolved lipidomics, based on capillary microsampling and liquid chromatography mass spectrometry (DAPNe-LC-MS). This method has been used in combination with PIXE imaging to probe the relationship between metals, metal-containing drugs, and lipids in discrete areas of diseased tissues. Metal accumulation is of interest in numerous diseases including Alzheimer’s, Parkinson’s, tuberculosis, and other infectious diseases, and therefore, this methodology should be of interest to researchers working across a range of disease and tissue types. We have shown that elemental imaging with spatially resolved lipidomics can be used to probe the spatial relationship between the element concentration (especially in this case, Fe) and lipids. We also show how drugs containing an elemental marker can be imaged using PIXE and that DAPNe-LC-MS can provide a confirmatory analysis.

## Supporting Information Available

Additional experimental details and results including microscopy images, EBS and PIXE maps, calibration lines, and statistical test results (PDF)
