# Supplementary material for: Colocation of Lipids, Drugs, and Metal Biomarkers Using Spatially Resolved Lipidomics with Elemental Mapping
Source: Anal Chem. 2022 Aug 18;94(34):11798–806. doi: 10.1021/acs.analchem.2c01940 (PMC9434551; doi:10.1021/acs.analchem.2c01940)
Supplement: Supplementary file 1 — ac2c01940_si_001.pdf [file ac2c01940_si_001.pdf]

Supporting Information

Co-location of lipids, drugs and metal biomarkers using spatially resolved lipidomics with elemental mapping

Holly-May Lewis[a], Catia Costa[b], Véronique Dartois[c] , Firat Kaya[c], Mark Chambers[d], Janella de Jesus[a], Vladimir Palitsin[b], Roger Webb[b] and Melanie J. Bailey[a*]

[a] Department of Chemistry, University of Surrey, Guildford, Surrey GU2 7XH, UK

[b] University of Surrey Ion Beam Centre, Guildford, Surrey GU2 7XH, UK

[c] Center for Discovery and Innovation, Hackensack Meridian School of Medicine, 123 Metro Blvd, Nutley, NJ 07110, USA

[d] Faculty of Health and Medical Sciences, University of Surrey, Guildford, Surrey GU2 7XH, UK

Table of Contents

Figure S1	Optical images of the granuloma areas probed by DAPNe
Figure S2	The P values to show which classes are significantly different between the regions of interest
Figure S3	Chemical structure of bedaquiline
Figure S4	Elastic backscattering spectrometry (EBS) map for TB granuloma showing the major element content of the tissue (C, N, O).
Figure S5	Calibration curve for bedaquiline from 0-20 ng/ml. Bedaquiline
Figure S6	Potassium PIXE map (2 x 2 mm2) before and after DAPNe extraction

	1	2	3
(A) Necrotic	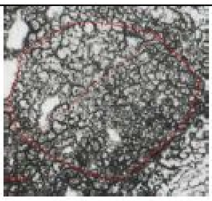	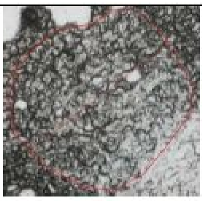	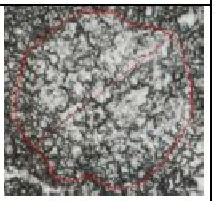
	166 μm<br>21100 μm^2	172 μm<br>22900 μm^2	142 μm<br>15200 μm^2
(B) Foamy macrophages (iron region)	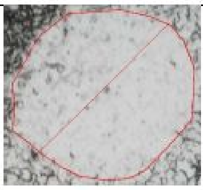	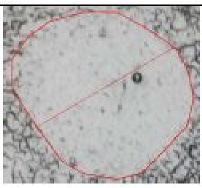	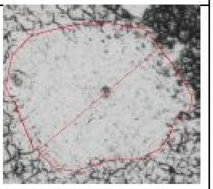
	138 μm<br>13900 μm^2	118 μm<br>12900 μm^2	135 μm<br>14700 μm^2
(C) Cellular rim (bromine region)	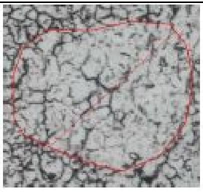	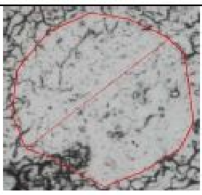	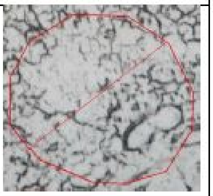
	127 μm<br>12400 μm^2	120 μm<br>10600 μm^2	105 μm<br>9400 μm^2
(D) Healthy	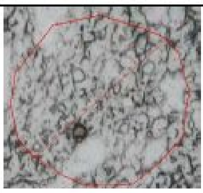	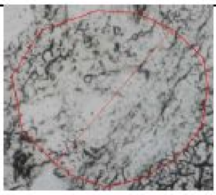	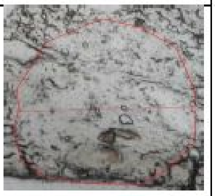
	140 μm<br>15300 μm^2	143 μm<br>17500 μm^2	173 μm<br>25100 μm^2

Figure S1. Optical images of the granuloma areas probed by DAPNe for (A) Necrotic region, (B) Foamy macrophage region, (C) Cellular rim region and (D) Healthy region, with triplicate extractions for each region

Ceramides	Caseum	FM	CR	Healthy
Caseum				
FM	> 0.05			
CR	> 0.05	> 0.05		
Healthy	> 0.05	< 0.05	> 0.05	

Monoglycerides	Caseum	FM	CR	Healthy
Caseum				
FM	> 0.05			
CR	> 0.05	> 0.05		
Healthy	> 0.05	> 0.05	> 0.05	

Diglycerides	Caseum	FM	CR	Healthy
Caseum				
FM	> 0.05			
CR	> 0.05	> 0.05		
Healthy	> 0.05	> 0.05	> 0.05	

Sphingoid bases	Caseum	FM	CR	Healthy
Caseum				
FM	> 0.05			
CR	< 0.05	< 0.05		
Healthy	> 0.05	> 0.05	< 0.05	

Fatty acids	Caseum	FM	CR	Healthy
Caseum				
FM	> 0.05			
CR	> 0.05	> 0.05		
Healthy	> 0.05	< 0.05	< 0.05	

Sphingomyelins	Caseum	FM	CR	Healthy
Caseum				
FM	> 0.05			
CR	> 0.05	> 0.05		
Healthy	> 0.05	> 0.05	> 0.05	

Phopholipids	Caseum	FM	CR	Healthy
Caseum				
FM	> 0.05			
CR	> 0.05	> 0.05		
Healthy	< 0.05	< 0.05	> 0.05	

Triglycerides	Caseum	FM	CR	Healthy
Caseum				
FM	> 0.05			
CR	> 0.05	> 0.05		
Healthy	> 0.05	> 0.05	> 0.05	

Figure S2. The P values to show which classes are significantly different between the regions of interest

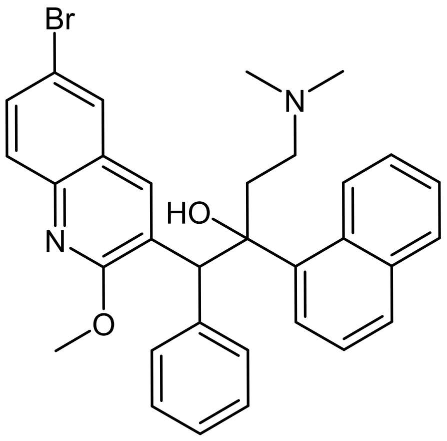

Figure S3. Chemical structure of bedaquiline

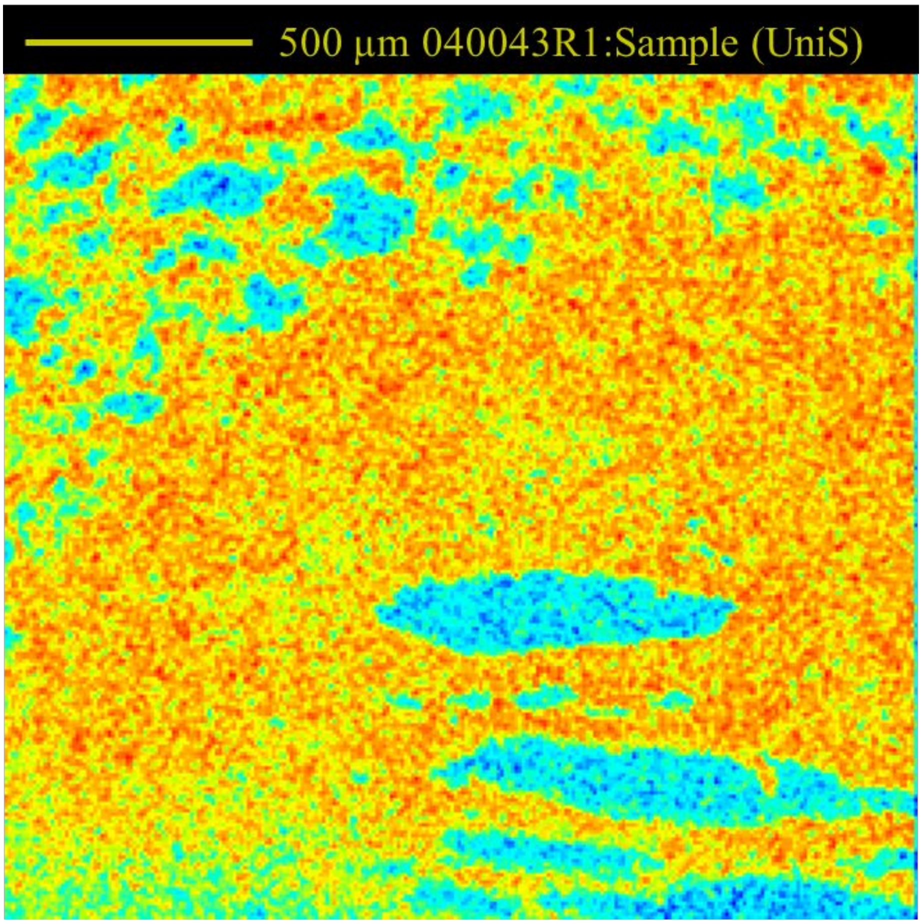

Figure S4. Elastic backscattering spectrometry (EBS) map for TB granuloma showing the major element content of the tissue (C, N, O).

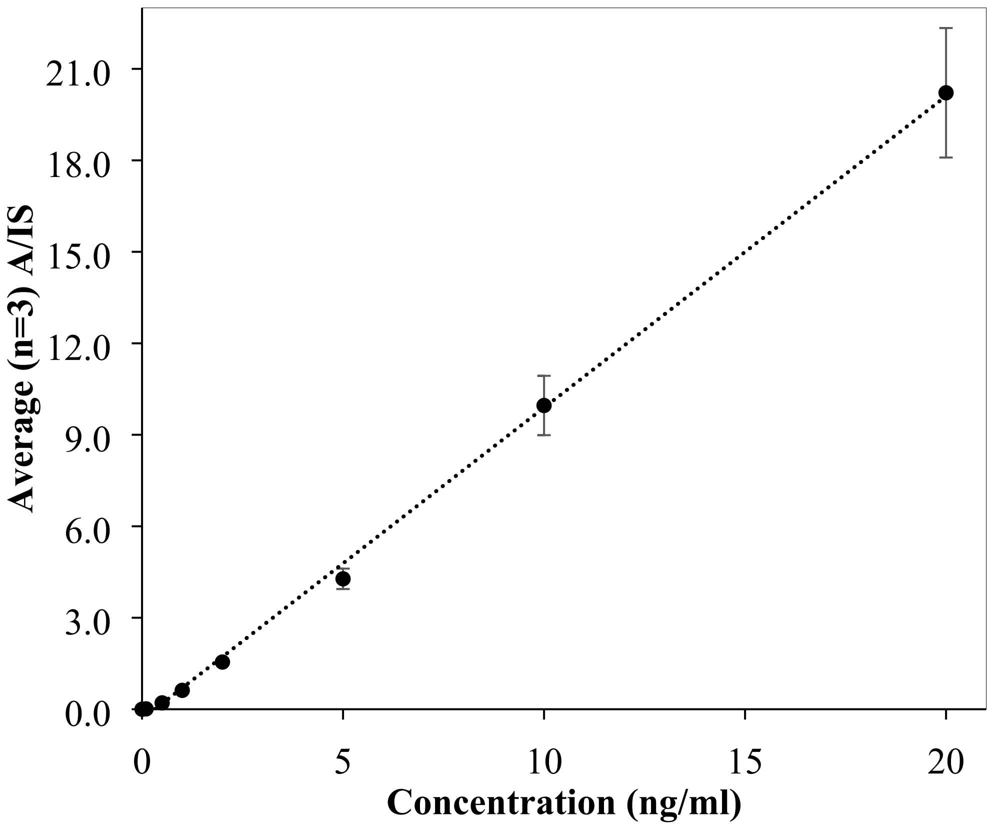

Figure S5. Calibration curve for bedaquiline from 0-20 ng/ml. Bedaquiline limit of detection was calculated as 2.5 pg.

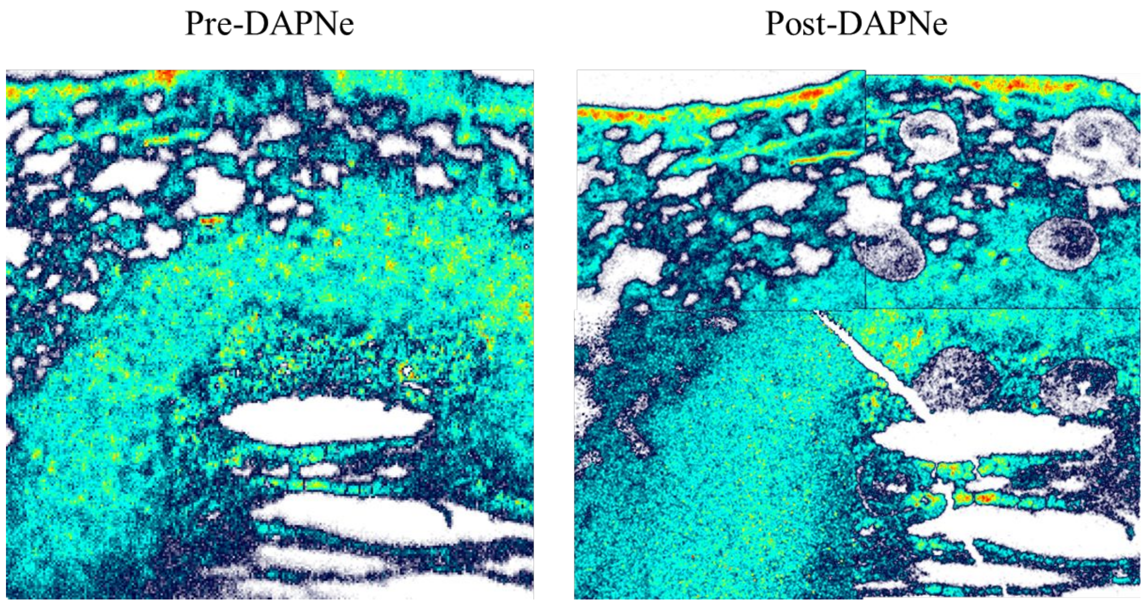

Figure S6. Potassium PIXE map (2 x 2 mm^2) before and after DAPNe extraction.
